# Magnetic resonance imaging of fetal persistent left superior vena cava

**DOI:** 10.1038/s41598-017-04591-y

**Published:** 2017-06-23

**Authors:** Su-Zhen Dong, Ming Zhu

**Affiliations:** Department of Radiology, Shanghai Children’s Medical Center, Shanghai Jiaotong University School of Medicine, Shanghai, 200127 China

## Abstract

This study aimed to evaluate the diagnostic accuracy of fetal magnetic resonance imaging (MRI) for persistent left superior vena cava (LSVC). Prenatal echocardiography (echo) and/or ultrasound (US) and MRI data for 49 fetuses with persistent LSVC, confirmed via postnatal diagnoses between January 2010 and October 2015, were retrospectively reviewed. All prenatal MRI was performed at 1.5 T. Imaging sequences included steady-state free-precession (SSFP), single-shot turbo spin echo (SSTSE), and other sequences. All 49 cases of fetal persistent LSVC were correctly diagnosed via MRI, but only 34 cases (69.4%) were correctly diagnosed via an initial US and/or echo before MRI. Of the 15 cases that were not correctly diagnosed via US and/or echo, 8 had congenital heart diseases (CHDs) and 7 were without CHDs; however, they were associated with extracardiac abnormalities or maternal obesity. Thirty-five cases were associated with other cardiovascular abnormalities; 8, with extracardiac abnormalities; and 6, with no associated condition. In 44 (89.8%) cases, the innominate veins were absent; the remaining cases had innominate veins. In 14.3% of patients (7 cases), the persistent LSVC drained directly into the atrium. Fetal MRI can detect persistent LSVC and play an adjunctive role along with US in the evaluation of persistent LSVC.

## Introduction

Persistence of thepersistent left superior vena cava (LSVC) is a common anomaly of the cardinal systemic venous drainage system. Persistent LSVC can occur in isolation or may be associated with other congenital heart diseases (CHDs). Its incidence rate is 0.3–0.5% in the general population and 4–8% in patients with CHD^1, 2^, but its true prevalence is not known because persistent LSVC usually has no clinical significance and hence remains undetected. However, a prenatal diagnosis of persistent LSVC is sometimes helpful because it may be associated with cardiac and extra-cardiac diseases with an incidence rate as high as 83% and 48%, respectively^1, 2^.

Prenatal US is the first choice to visualize fetal persistent LSVC. The role of fetal MRI as an adjunct tool to US has grown in the past several years^3–5^. Although several studies have documented the potential of MRI in diagnosing congenital heart defects^6–11^ and vascular abnormalities^12, 13^, the application of prenatal MRI to persistent LSVC has, to the best of our knowledge, not yet been reported.

In this study, we analyze the diagnostic accuracy of fetal MRI for prenatal persistent LSVC.

## Materials and Methods

### Study subjects

This is a retrospective review of the diagnosis of fetal persistent LSVC based on fetal MRI data collected between January 2010 and October 2015. The MRI studies were identified from the clinical fetal MRI database of 4668 cases. In all, 49 fetuses with persistent LSVC confirmed via postnatal imaging or operation or necropsy reports were evaluated using fetal echocardiography (and/or US) and fetal cardiovascular magnetic resonance (CMR) in our hospital. Termination of pregnancy was chosen by the parents in other 9 cases where persistent LSVC could not be confirmed by necropsy, and these cases were not included in this study. Detailed fetal US and/or echocardiography was usually performed within an average 1.5 (range, 1–2) days before the fetal MRI examination. MRI was performed for clinical indications after inconclusive or suspicious prenatal US or echocardiography results to confirm or possibly expand the diagnosis. MRI readers were not blinded to the outcomes of the fetal echocardiography examinations. Eight pregnant women were examined only via general fetal US; 41 patients were examined via US and fetal echocardiography.

### Magnetic resonance imaging

MRI was performed using two 1.5 T units (Signa Echospeed; GE Medical Systems, Milwaukee, WI, and Achieva Nova dual; Philips Medical Systems, Best, The Netherlands). Most examinations were carried out on the Philips® MR unit. Gestational age ranged from 20 to 35 weeks (mean, 24 weeks). The age of the pregnant women ranged from 20 to 39 years (mean, 29 years). A multiplanar steady-state free-precession (SSFP) sequence, non-gated cine SSFP sequence, and single-shot turbo spin echo (SSTSE) sequence were used to evaluate the anatomy and pathologies of each fetal heart. Only T1-weighted MR images were used to evaluate colon deformities identified among the extracardiac abnormalities. Fast T2-weighted MR images were acquired with the Philips® units using balanced turbo field echo (B-TFE) sequences, “real-time” B-TFE (B-TFE-RLT) sequences, and SSTSE sequences. All SSTSE sequences (TR/TE, 12000/80 ms; field of view, 260–355 mm^2^; section thickness, 6–10 mm; spacing, 0–0.5 mm; matrix, 172 × 173–236 × 220; flip angle, 90°) were acquired mainly in an oblique coronal view to show the bronchus and assess the visceroatrial situs. The B-TFE sequences (TR/TE, 3.6/1.8 ms; field of view, 260–325 mm^2^; section thickness, 4–6 mm; spacing, −2 to −4 mm; matrix, 172 × 173–216 × 218; flip angle, 80°) were mostly acquired in the transverse view of the fetal thorax; the four-chamber plane; the short-axis, coronal, and oblique sagittal planes of the fetal heart; and especially, the transverse view of the aortic arch and the four chambers. B-TFE-RLT sequences were acquired along the transverse and short-axis planes of the fetal heart. The total acquisition time was 20–25 min. No sedation, contrast media, or fetal cardiac gating were used.

The ethics committee of our institution approved the study. All pregnant mothers involved in the study provided written informed consent. All methods of the study were performed in accordance with the relevant guidelines and regulations.

## Results

**Figure 1:**
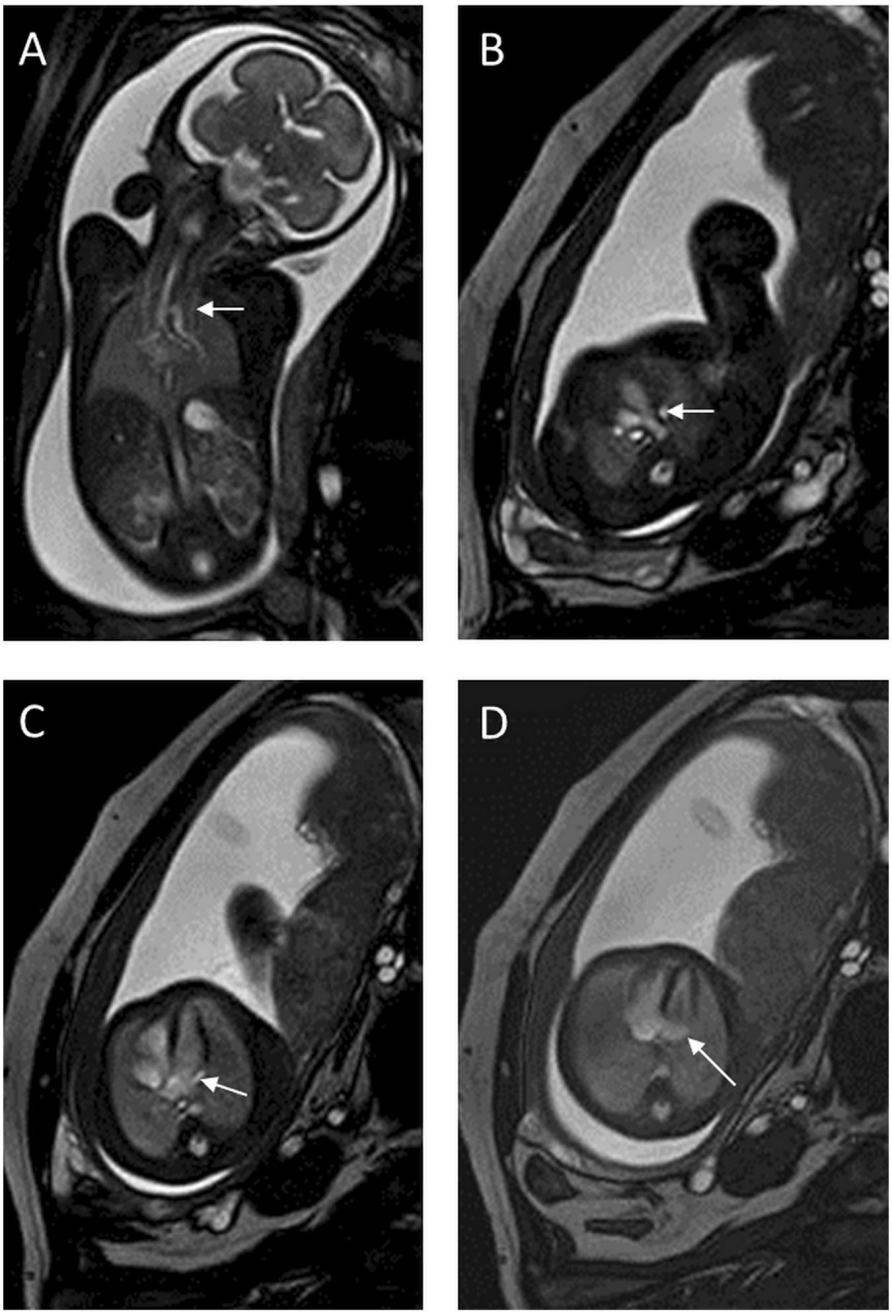
A 27-week fetus with persistent left superior vena cava. Fetal CMR B-TFE coronal and transverse views of the aortic arch show persistent left superior vena cava (LSVC) on the left side of the aortic arch (arrows in **A** and **B**); sequential four-chamber views show persistent LSVC at the back of the left atrium (arrow in **C**) and an enlarged coronary sinus (arrow in **D**).

We identified 49 cases (46 mothers with a single fetus and three with twins) of fetal persistent LSVC using fetal MRI, including 14 cases without other cardiovascular abnormalities (Fig. 1) and 35 cases with other cardiovascular abnormalities. In these 35 cases, other cardiovascular abnormalities were all confirmed via postnatal imaging or operation.

**Figure 2:**
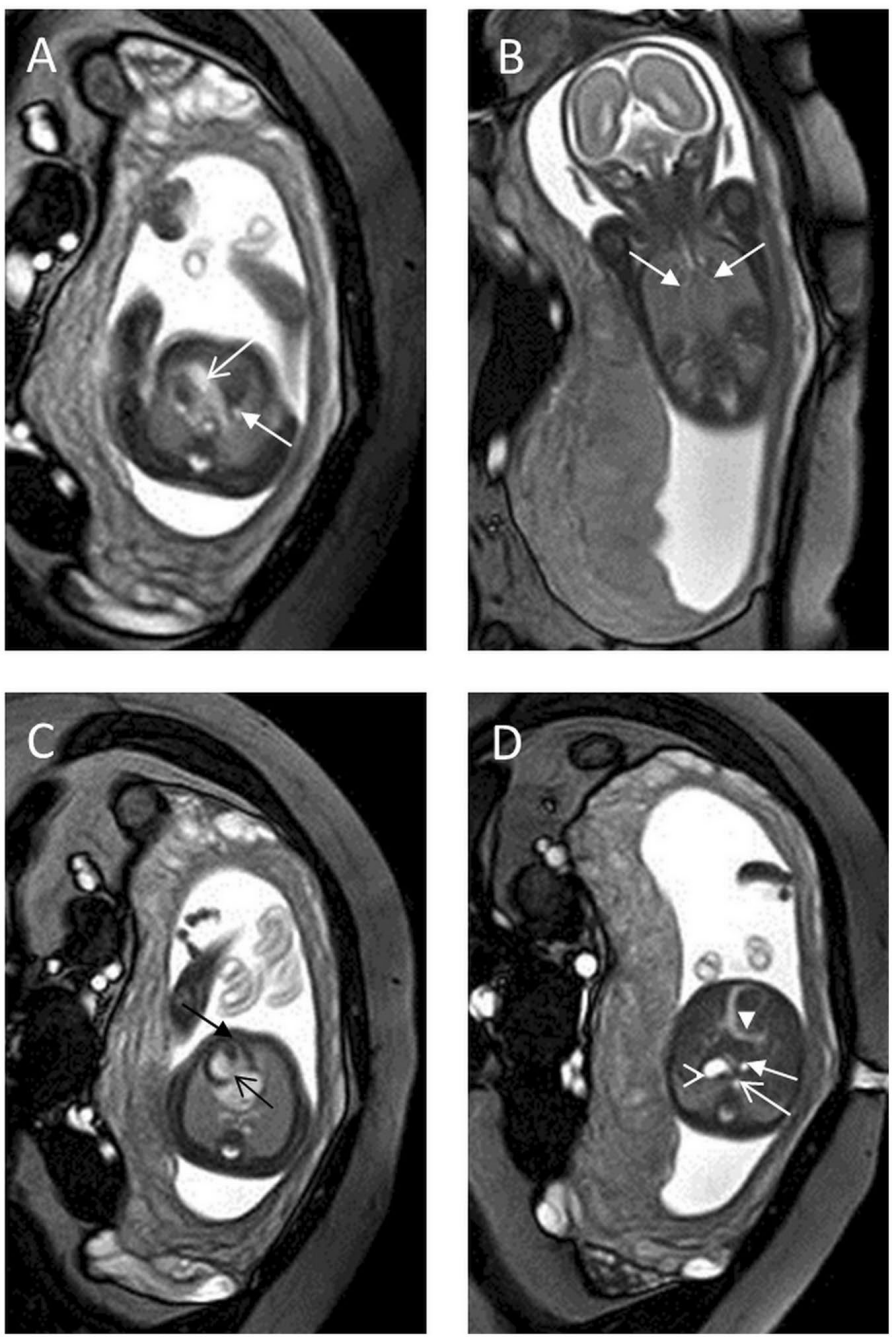
A 24-week fetus with asplenia syndrome and persistent LSVC. Fetal CMR B-TFE transverse view of the aortic arch shows persistent LSVC on the left side of the aortic arch (arrow in **A**) and a dilated aorta (open arrow in **A**); coronal view shows bilateral main bronchial symmetry (arrows in **B**); four-chamber and transverse views show dextrocardia (black arrow in **C**) and a right-sided stomach (open arrowhead in **D**), complete atrioventricular septal defect (black open arrow in **C**), a single ventricle (black arrow in **C**), the left descending aorta (DAo) (open arrow in **D**) and inferior vena cava (IVC) (arrow in **D**), and a midline liver (arrowhead in **D**).

**Figure 3:**
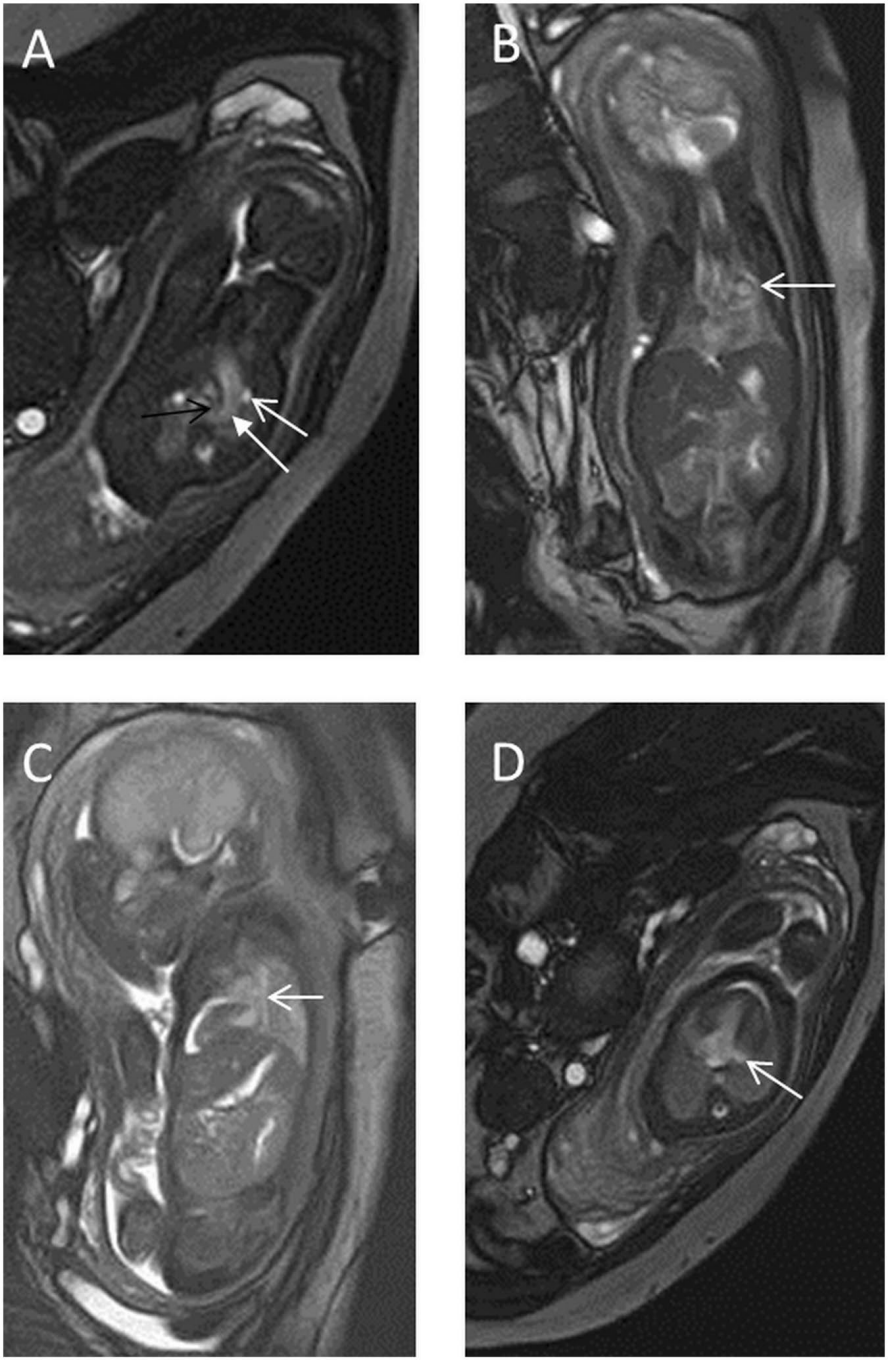
A 32-week fetus with coarctation of the aorta and persistent LSVC. Fetal CMR B-TFE transverse view of the aortic arch and coronal and oblique sagittal views show persistent LSVC (white open arrows in **A,B** and **C**), CoA (black open arrow in **A**) and a large ductus arteriosus arch (arrow in **A)**; four-chamber views show an enlarged coronary sinus (open arrow in **D**).

**Figure 4:**
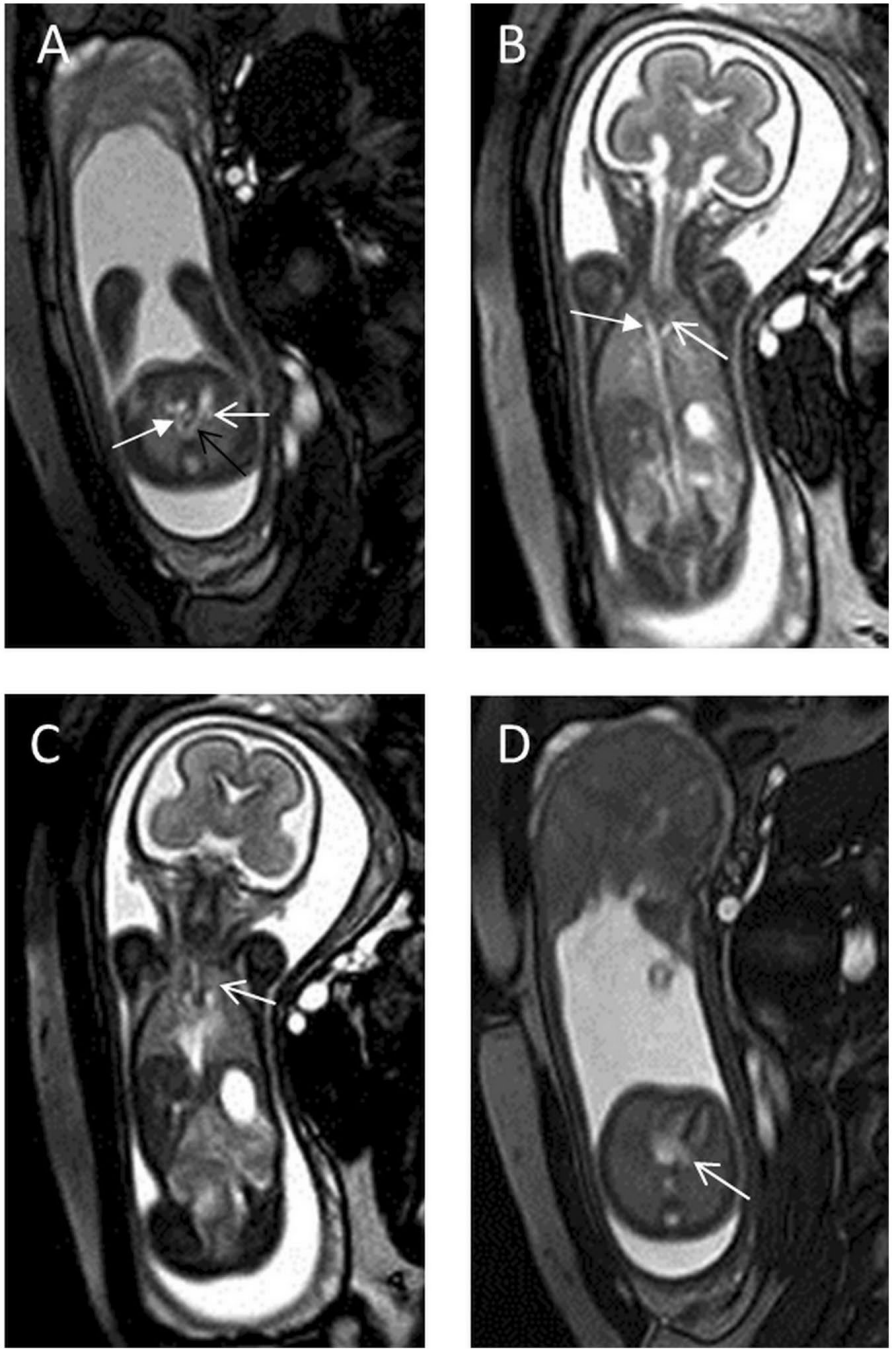
A 22-week fetus with right aortic arch with an aberrant left subclavian artery and persistent LSVC. Fetal CMR B-TFE transverse view of the aortic arch and coronal views show persistent LSVC (white open arrows in **A** and **C**) and the right aortic arch (arrows in **A** and **B**) with an aberrant left subclavian artery (black open arrow in **A** and white open arrow in **B**); four-chamber view shows an enlarged coronary sinus (open arrow in **D**).

The congenital cardiovascular abnormalities in the 35 fetuses included heterotaxy syndromes (n = 9) (8 cases of asplenia and 1 case of polysplenia) (Fig. 2), tricuspid atresia (n = 1), ventricular septal defects (VSDs, n = 5), double-outlet right ventricle (DORV, n = 3), complete transposition of great arteries (TGA, n = 2), coarctation of the aorta (CoA, n = 6) (Fig. 3), double aortic arch (n = 1), right aortic arch (RAoA) with vascular ring (n = 3) (Fig. 4), pulmonary atresia with ventricular septal defect (PA/VSD) (n = 1), Tetralogy of Fallot (TOF, n = 2), and hypoplastic left heart syndrome (HLHS, n = 2).

Of the 14 cases without associated structural heart disease, eight cases had extracardiac abnormalities, and the other six had persistent LSVC as an isolated finding. Three of the 8 mothers with fetuses that showed extracardiac abnormalities underwent medical termination of their pregnancy.

**Figure 5:**
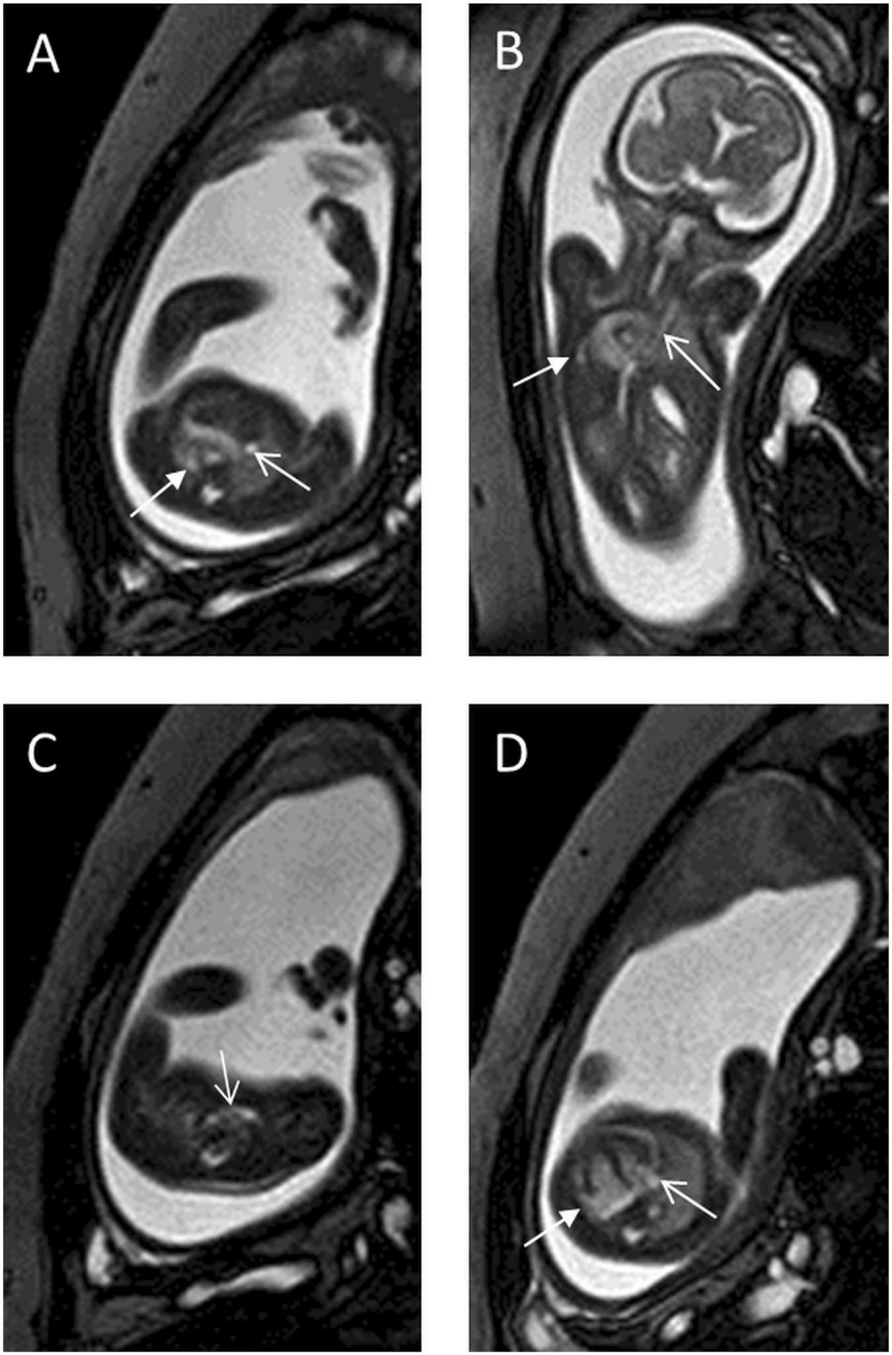
A 23-week fetus with right pulmonary hypoplasia and persistent LSVC. Fetal CMR B-TFE transverse, coronal and four-chamber views show persistent LSVC (open arrows in **A**,**B** and **D**), the right superior vena cava (arrow in **A**), a bridging vein connecting the left and right superior vena cava (open arrow in **C**) and a hypoplastic right lung (arrows in **B** and **D**).

The extracardiac abnormalities included agenesis of the corpus callosum with Dandy–Walker syndrome (n = 1), enlarged cisterna magna with left clubfoot (n = 1), right pulmonary hypoplasia (n = 1) (Fig. 5), gastroschisis (n = 1), bilateral renal dysplasia (n = 1), left multicystic dysplastic kidney disease (n = 1), cloacal dysgenesis (n = 1), and oligohydramnios (n = 1).

All 49 cases of fetal persistent LSVC were correctly diagnosed via fetal MRI, but only 34 cases (69.4%) were correctly diagnosed via fetal US and/or echocardiography. Of the 15 cases that were not correctly diagnosed via fetal US and/or echocardiography, 8 had congenital heart diseases and 7 were without congenital heart diseases; however, these 7 cases were associated with extracardiac abnormalities or maternal obesity. After fetal MRI examinations, all 15 cases again underwent fetal echocardiography, wherein 12 cases were found to have persistent LSVC, except for one fetus with oligohydramnios, one multiple pregnancy with twins, and one case of maternal obesity (Table 1).

**Table 1:** Reasons for inaccurate diagnosis by Ultrasound.

Reason	No. of Fetuses
Extracardiac abnormalities, maternal obesity or large gestational age, without CHD	7
Extracardiac abnormalities or twins, with CHD	3
Heterotaxy syndromes	4
No factors	1

**Figure 6:**
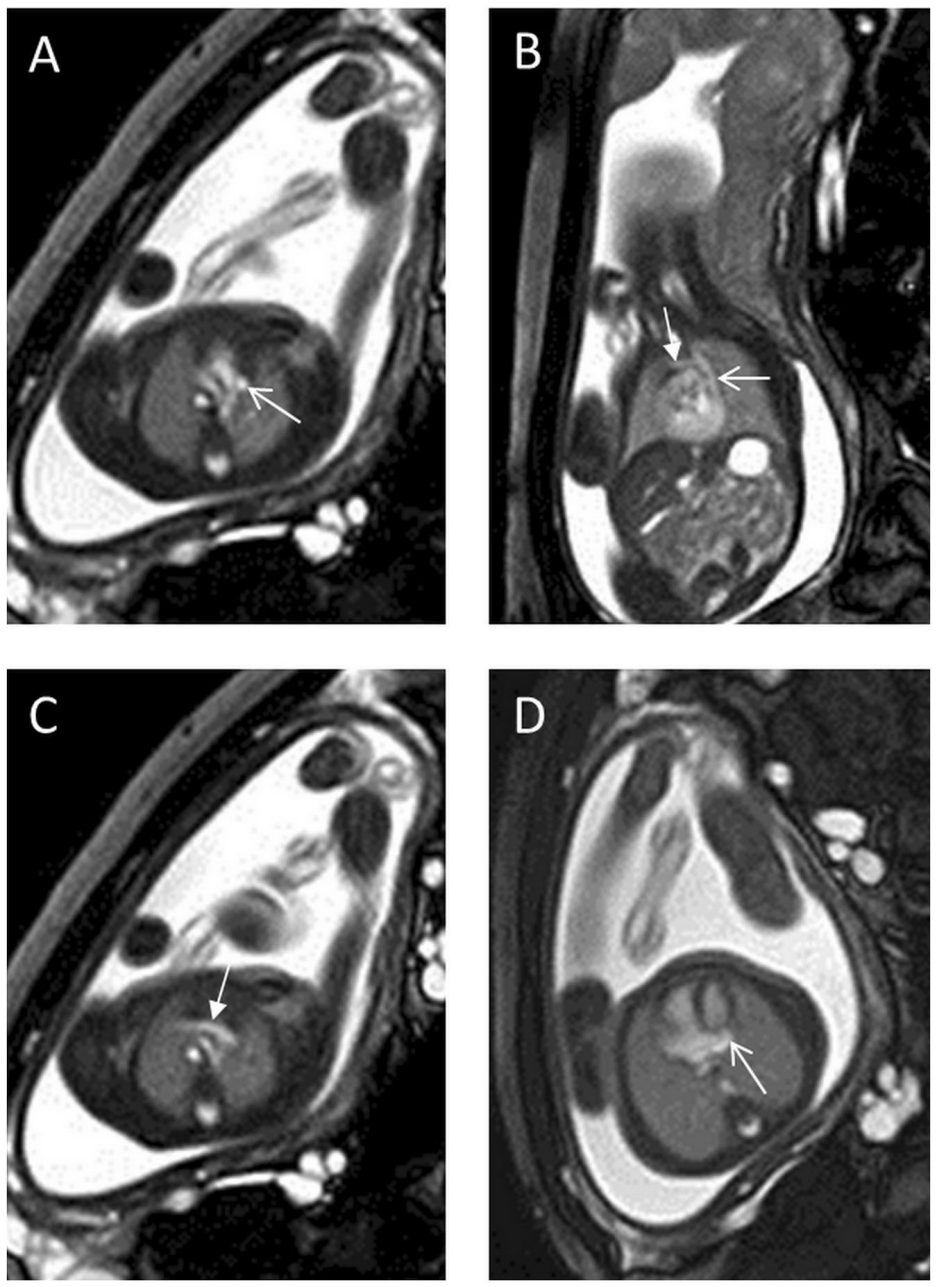
A 24-week fetus with absent right superior vena cava and persistent LSVC. Fetal CMR B-TFE transverse, oblique sagittal, and four-chamber views show the absence of the right superior vena cava, persistent LSVC (open arrows in **A** and **B**), a bridging vein (arrows in **B** and **C**), and an enlarged coronary sinus (open arrow in **D**).

One fetus had no right superior vena cava (RSVC) (Fig. 6); all of the remaining cases had a normal RSVC. In 44 (89.8%) cases, the innominate veins were absent. The remaining cases had innominate veins that were correctly identified via fetal MRI but not echocardiography. In 7 (14.3%) cases, the fetal persistent LSVC drained directly into the atrium. In 42 (85.7%) cases, it drained into the coronary sinus, resulting in its enlargement.

SSFP imaging of the transverse view of the aortic arch is adequate for diagnosis. Transverse views of the aortic arch from MR SSFP imaging clearly showed persistent LSVC with hyperintensity, which was indicated by a vessel visible on the left side of the aortic arch in all 49 cases.

## Discussion

The LSVC is a persistent remnant of a vessel that normally disappears; it is the residual proximal section of the left anterior cardinal vein^1, 2, 14, 15^. A persistent LSVC typically runs down the back of the left atrium and enters the right atrium through the orifice of an enlarged coronary sinus. Persistent LSVC represents the most common variation in the systemic venous return system^1, 2, 14^ and is common among patients with CHDs^1, 2, 14, 15^. In this study, 71.4% (35/49) of cases were associated with other cardiovascular abnormalities.

Unlike prenatal US, MRI is unaffected by maternal and fetal conditions such as obesity, uterine myoma, twin pregnancies, and oligohydramnios^3–5, 16^. The usual timing of fetal echocardiography for the premature diagnosis of congenital heart disease is at approximately 20–26 weeks of gestation^7^. During the later phases of gestation, the development of certain conditions (e.g., relative reduction of the amniotic fluid volume and intensification of calcification in the ribs) may impair the quality of fetal echocardiography results^3–5, 7, 17^. In contrast, fetal MRI gives clearer results in older fetuses. When compared to echocardiography, fetal MRI does have some limitations: echocardiography can highlight certain defects, such as VSD, better than MRI^7^.

Transverse views of the aortic arch from MR imaging clearly showed persistent LSVC, which was indicated by a vessel visible on the left side of the aortic arch in all 49 cases. Although an anomalous pulmonary venous connection and the sub-aortic left innominate vein may have had a similar manifestation in transverse views of the aortic arch, persistent LSVC is more common, and it can also be visualized in axial MR images examined slice by slice in a continuous set of images; further, persistent LSVC can be traced into an enlarged coronary sinus. Persistent LSVC usually has no clinical implications because the systemic venous blood continues to return to the right atrium via the coronary sinus. Persistent LSVC has a greater prevalence with associated CHDs. Therefore, if persistent LSVC is first detected, other underlying CHDs will be carefully checked for in further examinations.

In this study, 49 cases of fetal persistent LSVC were correctly diagnosed via MRI, but only 34 cases (69.4%) were correctly diagnosed via fetal US and/or echocardiography at the first examination. Only 5 (10.2%) cases had an innominate vein. Fetal persistent LSVC is not very difficult to diagnose using fetal echocardiography. The “three-vessel view” of fetal echocardiography can show fetal persistent LSVC. Significant dilatation of the coronary sinus, which is best visualized in a four-chamber view, is also an indirect sign of persistent LSVC. However, the coronary sinus is unroofed in most cases of heterotaxy syndrome and will therefore not appear dilated on prenatal echocardiography^18^. Accordingly, persistent LSVC in cases of heterotaxy syndrome is not easy to find. In our study, 4 of 9 persistent LSVCs associated with heterotaxy syndrome were not detected in the first echocardiography. Seven cases with extracardiac abnormalities, maternal obesity or advanced gestational age were examined only via general US and not echocardiography before fetal MRI was performed. Typically, sonographers focus on extracardiac abnormalities and ignore persistent LSVC. Alternatively, owing to the presence of extracardiac abnormalities, such as oligohydramnios and gastroschisis, maternal obesity, or advanced gestational age (35 weeks gestation), fetal persistent LSVC cannot always be accurately diagnosed. Two cases were associated with extracardiac abnormalities (e.g., congenital diaphragmatic hernia and bilateral renal dysplasia) and one case involved twins and CHD; both scenarios affected the accurate diagnosis of persistent LSVC and CHD. Only one case with CHD was incorrectly diagnosed upon undergoing fetal echocardiography by experienced doctors at our hospital because fetal echocardiography was performed for the first time at 34 weeks of gestation (Table 1).

The transverse view of the aortic arch in fetal MRI is very easy to obtain, easy to reproduce, and easy to interpret. The axial view of the aortic arch on MRI is as important as the four-chamber view, as is the case for the three-vessel view in fetal echocardiography^18, 19^. Fetal persistent LSVC could be easily detected in the transverse view of the aortic arch. An extra vessel was mostly found on the left side of the aortic arch. Another significant indirect diagnostic sign was a dilated coronary sinus that could be visualized in the four-chamber view in fetal MRI. Usually, SSFP imaging of the transverse view of the aortic arch is adequate for diagnosis. With SSFP imaging, the vessels and blood pool in utero are visualized as hyperintense structures, which results in better delineation of cardiac structures than with SSTSE sequences^13^.

This study had some limitations. We did not study the number of cases with aneuploidy among the 49 fetuses with fetal LSVC. Moreover, we were unaware of the presence of any cases that may be undetected using both MRI and echocardiography. This was a retrospective study and is therefore subject to limitations associated with the study design. MRI readers were not blinded to the outcomes from fetal echocardiography examinations since these findings prompted the initial referral for fetal MRI. In addition, given that the aim of this study was not to compare the accuracy of fetal echocardiography and magnetic resonance imaging diagnoses, information regarding exact cardiac diagnoses is not included.

## Conclusions

Fetal MRI can detect persistent LSVC and play an adjunctive role along with US in the evaluation of persistent LSVC. Fetal MRI can also display the innominate vein between the bilateral superior vena cava.
